# Mycotoxins-Imprinted Polymers: A State-of-the-Art Review

**DOI:** 10.3390/toxins16010047

**Published:** 2024-01-15

**Authors:** Simone Cavalera, Laura Anfossi, Fabio Di Nardo, Claudio Baggiani

**Affiliations:** Laboratory of Bioanalytical Chemistry, Department of Chemistry, University of Torino, 10125 Torino, Italy; simone.cavalera@unito.it (S.C.); laura.anfossi@unito.it (L.A.); fabio.dinardo@unito.it (F.D.N.)

**Keywords:** mycotoxin, molecularly imprinted polymer, mimic template, solid-phase extraction, magnetic solid-phase extraction, stir bar sorptive extraction, food analysis

## Abstract

Mycotoxins are toxic metabolites of molds which can contaminate food and beverages. Because of their acute and chronic toxicity, they can have harmful effects when ingested or inhaled, posing severe risks to human health. Contemporary analytical methods have the sensitivity required for contamination detection and quantification, but the direct application of these methods on real samples is not straightforward because of matrix complexity, and clean-up and preconcentration steps are needed, more and more requiring the application of highly selective solid-phase extraction materials. Molecularly imprinted polymers (MIPs) are artificial receptors mimicking the natural antibodies that are increasingly being used as a solid phase in extraction methods where selectivity towards target analytes is mandatory. In this review, the state-of-the-art about molecularly imprinted polymers as solid-phase extraction materials in mycotoxin contamination analysis will be discussed, with particular attention paid to the use of mimic molecules in the synthesis of mycotoxin-imprinted materials, to the application of these materials to food real samples, and to the development of advanced extraction methods involving molecular imprinting technology.

## 1. Introduction

Mycotoxins are secondary metabolites produced by many fungal species. These natural toxicants are of concern in terms of food contamination and have long been known to be a significant source of food-borne illness posing severe risks to human health, not only after a single massive exposure, but, more often, after continuous exposure to low doses, and that such exposure can be related to several chronic diseases, including some types of cancer and serious hormonal dysfunctions [1,2,3]. 

For mycotoxins, analytical methods characterized by a high sensitivity and selectivity, rapidity of execution, low cost, and suitability for mass screening are required worldwide [4,5]. Commercially available rapid mass screening assays based on the use of immunoanalytical techniques are widely diffused [6,7], but very frequently, a mycotoxin-contaminated sample must be confirmed by a separative analytical technique coupled with a mass spectrometry detector [8,9]. These instrumental techniques are sufficiently sensitive for the detection and quantification of mycotoxin contamination, but direct application on real samples is difficult and a quantitative analysis can be performed only after extensive clean-up and preconcentration steps [10]. Moreover, concerning current sample pre-treatment methods, they are based mostly on solid-phase extraction (SPE) techniques that are fast and cheap but not selective, while methods based on immunoaffinity extraction are very selective but expensive. Thus, economical, rapid, and target-focused extraction and purification approaches based on innovative solid-phase supports are required [11,12,13]. 

Molecularly imprinted polymers are synthetic materials whose main feature is the presence of artificial binding sites which selectively recognize a target molecule [14,15,16]. These materials are obtained via polymerization around a template molecule of one or more functional monomers selected according to their capacity to interact with the functional groups of the template. The presence of a cross-linking agent in the pre-polymerization mixture assures the formation of a stable three-dimensional network polymer containing binding sites with shape, size, and functional groups complementary to the template molecule. Once the template is removed, the artificial binding sites show the same properties of antibodies, i.e., similar thermodynamic and kinetic behavior, reversible binding, marked selectivity, and a high affinity constant.

**Figure 1:**
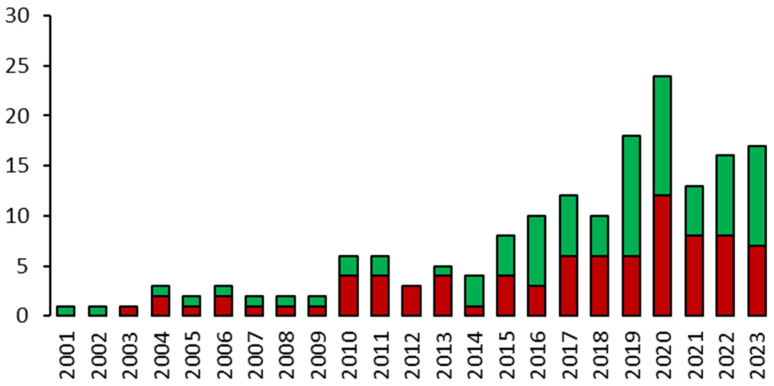
Works published annually about molecular imprinting of mycotoxins. Red bars: molecularly imprinted solid phase extraction. Green bars: other related topics. Year 2023 limited to September included (Web of Science, Retrieved 2 October 2023, https://www.webofscience.com/wos/woscc).

Molecularly imprinted polymers represent good candidates for circumventing the aforementioned defects [17]. In fact, in recent years, there has been a progressive increase in the literature concerning the development and use of MIPs with molecular recognition properties towards mycotoxins (Figure 1). In this review, after an overview of the approaches for efficiently developing MIPs’ sorbents towards mycotoxins, the state-of-the-art about their application in the analysis of food contamination will be discussed.

## 2. Drawbacks and Remedies in Mycotoxin Imprinting

Compared with other imprinting targets, mycotoxins are more difficult templates, but it must be considered that such issues do not arise from a lack of functional groups suitable for establishing non-covalent interactions during the imprinting process. In fact, the molecular structures of mycotoxins present many polar groups suitable for hydrogen bonds or ion–pair interactions with functional monomers, and they are fully compatible with the reagents—i.e., solvents, functional monomers, cross-linkers, and radical initiators—used in molecular imprinting technology [18]. Rather, due to the high toxicity of many mycotoxins and the risk of long-term effects, it can be difficult to directly manipulate the amounts of mycotoxins required to prepare a quantity of imprinted polymer suitable to set-up a solid-phase extraction protocol. Another drawback concerns their commercial accessibility, as, while companies selling analytical standards of mycotoxins are widespread, it is difficult to purchase bulk amounts of the same toxins or related minor metabolites (e.g., ochratoxin B, aflatoxin M1, and zearalenols) at an affordable price. Moreover, when MISPE protocols for mycotoxins are developed, the main critical point concerns the template not being removed completely from the imprinted polymer, thus slowly leaking during the following load, wash, and elution steps of the solid-phase extraction protocol and releasing little by little subsequently, interfering with the analysis process. Such template slow release (“bleeding”) is frequently detected at trace levels during the elution step, is difficult to eliminate completely, and represents a significant source of interference and systematic error in trace analysis [19,20].

**Figure 2:**
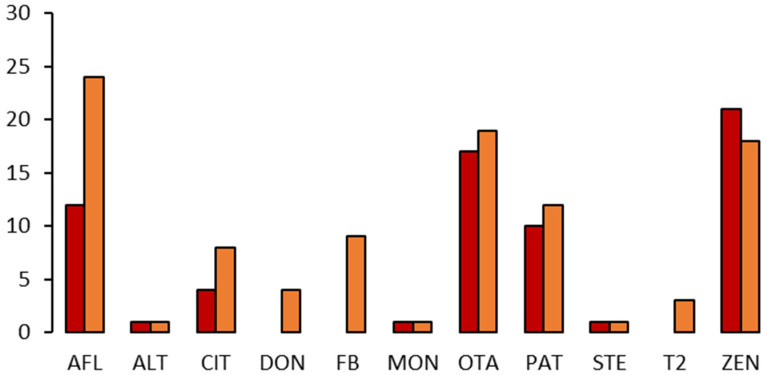
Templates used to prepare mycotoxin-imprinted polymers. Red bars: mimic/fragmental templates. Orange bars: polymers imprinted with target mycotoxins. AFL: aflatoxins; ALT: alternariols; CIT: citrinin; DON: deoxynivalenol; FB: fumonisins; MON: moniliformin; OTA: ochratoxin A; PAT: patulin; STE: sterigmatocystin; T2: T2-toxin; and ZEN: zearalenone; (Web of Science, Retrieved 2 October 2023, https://www.webofscience.com/wos/woscc). Molecular structures of mycotoxins are reported in Figure S1.

The drawbacks reported here can make the development of a mycotoxin-imprinted polymer difficult, but nevertheless, it is possible to use some approaches that make it possible to avoid them. In particular, an accurate choice of the molecular structure of the template in the “mimic template”, “fragmental template”, and “solid-phase polymer synthesis” approaches has proven effective [21]. At present, despite there being numerous examples of polymers being directly imprinted using the analytical targets as they are, a significant number of mycotoxin-imprinted polymers have been obtained using such approaches, which prove nearly equivalent in the case of ochratoxin A, zearalenone, and patulin (Figure 2).

### 2.1. The Template Mimic Approach

The “mimic template” approach was introduced for the first time more than twenty years ago [22]. This approach potentially solves the template bleeding problem through the involvement of a structural analogue related to the template molecule. The mimicking structure must be similar to the target analyte in such a way to elicit imprinted binding sites with good selectivity towards the latter, but it must also be structurally different in order to be clearly discriminated from the target molecule during the analytical separation performed after the extraction step. Thus, structural differences between the target analyte and the mimic template should be minimal and far from the substituents directly involved in non-covalent interactions with the binding sites.

In regard to mycotoxin imprinting, the mimic template approach has its main drawback in the difficulty in attaining some templates due to complexity of synthesis, high costs, or non-availability on the market. Thus, it may be necessary to replace templates with mimicking molecules with significant structural differences to the target analyte but that are more easily obtainable, where their similarity remains confined to their overall molecular shape and the preservation of substituents able to form non-covalent interactions with the binding sites.

**Figure 3:**
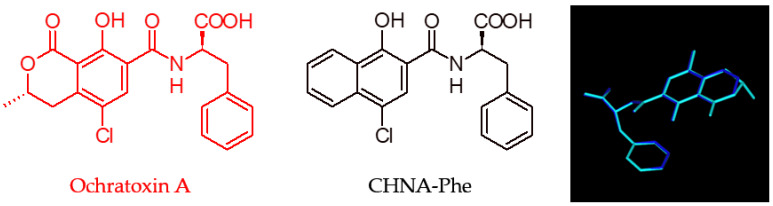
Molecular structures of OTA and its mimic, CHNA-Phe. In the insert: the superposition of OTA (blue) with CHNA-Phe (cyan).

The oldest example of the mimic template approach in mycotoxin imprinting is for ochratoxin A, where the substitution of the α-unsaturated lactone moiety with a naphthalene structure was deemed necessary in order not to over-complicate the synthesis and reduce its toxicity. The mimic template (N-(4-chloro-1-hydroxy-2-naphthoylamido)-(L)-phenylalanine, CHNA-Phe, Figure 3) preserved the general molecular structure of the target analyte, including the chirality of the L-phenylalanine substituent, the planarity of the benzopiranic sub-structure, the amide bridge, and the phenolic hydroxyl. A computational study showed an almost complete overlapping of ochratoxin A and CHNA-Phe, with a high level of molecular similarity in terms of solvent accessibility, electrostatic potential, and lipophilic/hydrophilic surfaces [23]. It was later observed that the structure of the mimic template fully determines the molecular recognition properties towards the target analyte, as polymers synthetized with the same mimic template but with different functional monomers presented the same recognition properties towards ochratoxin A [24,25,26].

**Figure 4:**
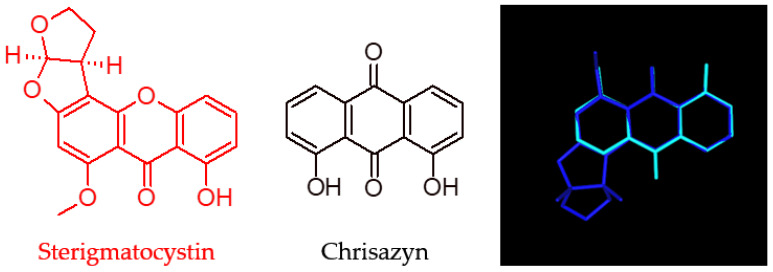
Molecular structures of STE and its mimic, chrisazin. In the insert: the superposition of STE (blue) with chrisazin (cyan).

As in the case for ochratoxins, sterigmatocystin (STE) presents a molecular structure characterized by a rigid bicyclic skeleton with adjacent hydroxylic (phenol) and carbonylic (quinone) substituents, suitable for hydrogen bond interactions with functional monomers (Figure 4). Thus, a mimic template able to retain the anthraquinone sub-structure was conceived, omitting the adjacent fused tetrahydrofuranic rings [27]. Chrisazin (1,8-dihydroxyanthraquinone) was used as a commercially available template in the preparation of an ormosil-based imprinted material, resulting in fluorescent microparticles with good binding properties towards the target template.

**Figure 5:**
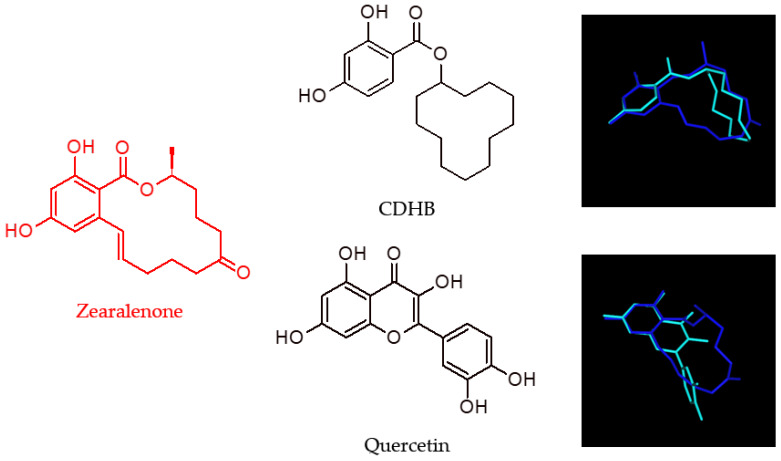
Molecular structures of ZEN and its mimics, CDHB and quercetin. In the inserts: the superposition of ZEN (blue) with CDHB and quercetin (cyan).

Aside from its toxicity, the direct use of zearalenone for the preparation of an imprinted polymer was found to be problematic due to the poor binding capacity of the resulting material, probably due to the covalent inclusion of the template in the polymer matrix induced by the presence of a double bond [28]. This drawback was overcome by Urraca et al. by using a mimic template (cyclododecanyl-2,4-dihydroxybenzoate, CDHB, Figure 5) coming from the esterification of resorcilic acid with cyclododecanol [29,30]. The mimic template was found to be easily synthesized in two steps, and its molecular structure preserved in their proper positions the key features of the target analyte: the two phenol groups in the meta position and the carbonyl group in ortho, while the 12-membered macrocyclic alcohol preserved the shape of the 14-membered macrocycle. Afterwards, Gadzala-Kopciuch et al., through a full physicochemical characterization of this mimic template, including a detailed crystallographic analysis and comparative molecular modelling, clarified that, although the cyclodedecyl ester is structurally different from zearalenone, its great conformational flexibility leads it to occupy the same space occupied by the latter, thus contributing with the same steric hindrance to generating binding sites significantly complementary to the target molecule. [31]. 

As an alternative to the use of ad hoc designed templates, molecules of natural origin have been used as template mimics of zearalenone. Quercetin was used in the preparation of magnetic imprinted particles based on an Fe_3_O_4_@SiO_2_ core covered by a layer of polystyrene with 4-vinylpyridine as a functional monomer through a multistep swelling synthetic procedure. Despite significant structural differences between the target analyte and mimic template, thanks to the presence of two phenol groups in the meta position and the carbonyl group in ortho onto the benzopyrone skeleton, the latter has been proven capable of generating 900 nm sized core-shell particles selective towards zearalenone, zearalenone, and α-β-zearalenols, while mycotoxins with different molecular structures (e.g., T2-toxin and deoxynivalenol) were not recognized [32,33].

**Figure 6:**
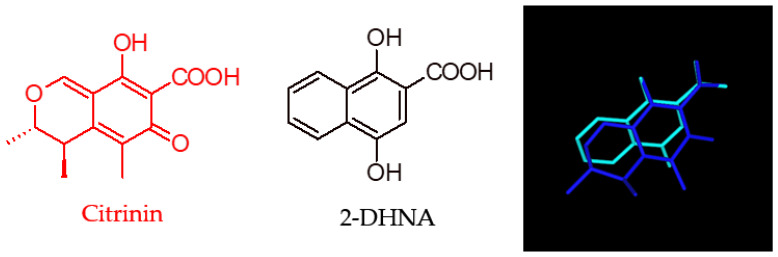
Molecular structures of CIT and its mimic, 2-DHNA. In the insert: the superposition of CIT (blue) with 2-DHNA (cyan).

Similar to the case of ochratoxin A, naphthalene was considered to be a suitable scaffold for building a mimic template for citrinin (Figure 6). Two different mimic templates, 1,4-dihydroxy-2-naphtoic acid (2-DHNA) and 1-hydroxy-2-naphtoici acid (2-HNA), have been reported in the literature separately [34,35]. Despite the absence of substituents that differentiate them from the target analyte, in both cases, the mimic templates proved capable of generating polymers with good molecular recognition capabilities towards the target analyte. Density functional calculations on the electronic structures suggest that the leading interaction driving the molecular recognition is based on the hydrogen bond between the carboxylic substituent on the naphthalene ring, the phenolic hydroxyl in position 1, and the 2-dimethylaminoethylmethacrylate used as a functional monomer, and that the strength of this interaction is nearly the same for both citrinin and the mimic templates.

**Figure 7:**
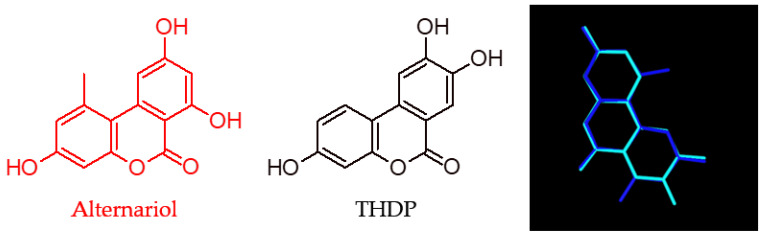
Molecular structures of AOH and its mimic THDP. In the insert: the superposition of AOH (blue) with THDP (cyan).

The identification of a suitable mimic template for the polyphenolic mycotoxin alternariol (Figure 7) required the synthesis of four different molecules, each of these characterized by a different number of phenolic groups in various positions and a different degree of O-methylation onto the dibenzo[b,d]pyran-6-one skeleton [36,37]. Different imprinted polymers were prepared with the mimic templates by using a library of basic, acidic, or neutral functional monomers, with divinylbenzene or ethyleneglycol dimethacrylate as cross-linkers. The binding screening showed that an imprinted polymer prepared with 3,8,9-trihydroxydibenzo[b,d]pyran-6-one (THDP) as a mimic template, N-(2-aminoethyl)methacrylamide as a functional monomer, and ethylene glycol dimethacrylate as a cross-linker was able to selectively bind the target analyte alternariol and its 9- and 7-monomethylether derivatives. 

**Figure 8:**
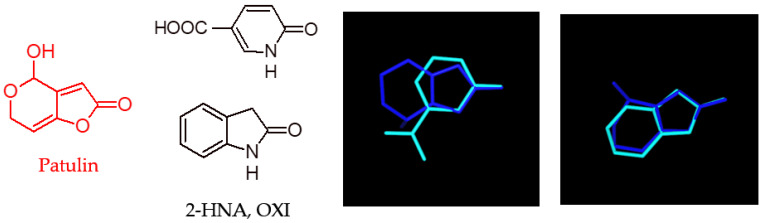
Molecular structures of PAT and its mimics, 2-HNA and OXI. In the inserts: the superposition of PAT (blue) with 2-HNA and OXI (cyan).

The synthesis of a mimic template for patulin (Figure 8) presents significant difficulties, as this mycotoxin is characterized by a particularly simple molecular structure, and therefore is not very suitable for modifications that do not alter its shape and properties; however, this is accompanied by a notable electrophilic reactivity coinciding with the opening of the lactone ring [38]. For this reason, many authors have chosen to use molecular structures only weakly related to patulin, such as 2-hydroxynicotinic acid (2-HNA) [39,40,41] or oxindole (OXI) [42,43,44,45], as template mimics. Despite their significant structural differences, these mimic templates were shown to be able to originate polymers with good binding properties towards the target molecule, and to be usable for solid-phase extraction applications.

**Figure 9:**
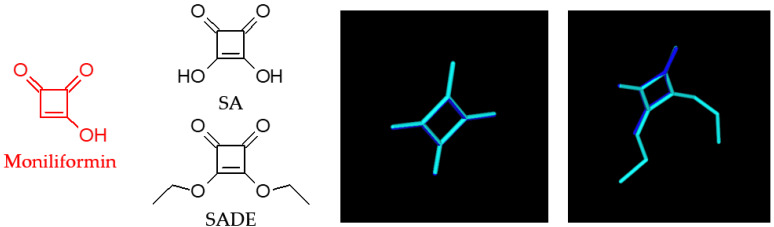
Molecular structures of MON and its mimics, SA and SADE. In the inserts: the superposition of MON (blue) with SA and SADE (cyan).

Moniliformin (Figure 9) is characterized by an exceptionally simple molecular structure if compared with all other known mycotoxins. In fact, it consists, in the anionic form, of the deoxysquaric acid. For this reason, the design of a mimic template necessarily requires the modification of a structure that is, in itself, very simple. Appell et al. solved the problem by using two commercially available structures, squaric acid and the corresponding diethyl ester [46]. Both the mimic templates were shown to be able to originate polymers with good binding properties towards moniliformin in organic polar solvents (acetonitrile, dimethylformamide, ethanol, and methanol) and in an acetonitrile–water mixture. 

### 2.2. The Fragmental Template Approach

The selection of a mimic template requires a certain level of creativity, but suitable structural modifications of the target molecule can be very difficult, expensive, or simply the target molecule has a structure too complex or unstable to be modified. In these cases, the mimic template strategy is brought to its extreme consequence in the fragmental imprinting approach, introduced in 2004 [47]. In this approach, the template consists of a structure markedly diverging from the target analyte as a whole, but similar to one of the sub-structures that compose the target analyte. Therefore, molecular recognition is guaranteed by the presence of appropriate functional groups positioned in the same way both on the fragment and on the target analyte.

**Figure 10:**
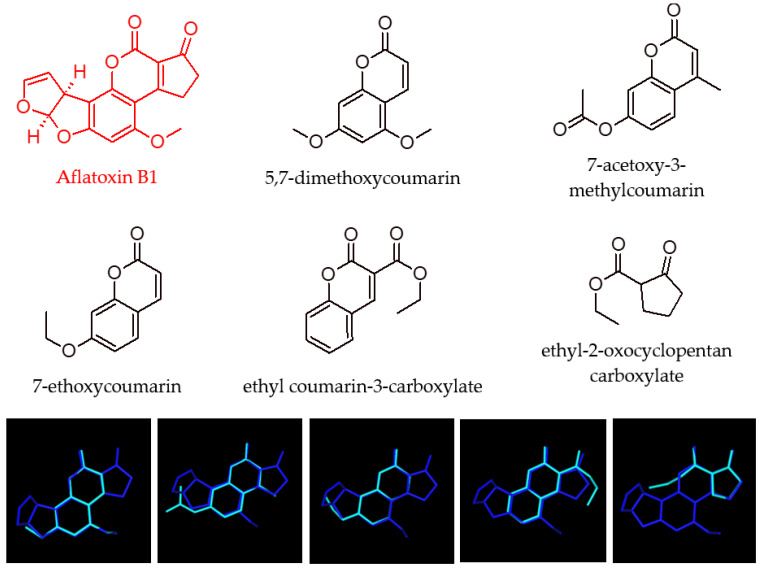
Molecular structures of AFB1 and its fragmental mimics. In the inserts: the superposition of AFB1 (blue) with (left to right, cyan): 5,7-dimethoxycoumarin, 7-acetoxy-3-methylcoumarin, 7-ethoxycoumarin, ethyl coumarin-3-carboxylate, and ethyl-2-oxocyclopentanecarboxylate.

Aflatoxins represent an emblematic case of the use of the fragmental approach. These mycotoxins, in addition to being significantly toxic and relatively expensive, have a peculiar molecular structure, for which it is rather difficult to identify similar substances capable of acting as mimic templates. Thus, in 2012, Wyszomirski and Prus proposed a computational approach for aflatoxin B1 involving 5,7-dimethoxycoumarin as a fragmental template [48]. After a molecular dynamic simulation, it turned out that methacrylic acid was able to establish five hydrogen bonds in the same positions for the template and the target aflatoxin, with nearly identical interaction energies. Afterwards, the validity of coumarins as fragmental templates was confirmed by several authors not only for 5,7-dimethoxycoumarin [49,50,51,52,53], but also for 7-acetoxy-3-methylcoumarin [54], 7-ethoxycoumarin [55], and ethylcoumarin-3-carboxyate [56] (Figure 10). It worth nothing that all the coumarins reported as templates preserve nearly the same structural motif characteristic of aflatoxins: the central coumarin-like structure (rings C and D). In a different computational approach, a non-coumarinic molecule, ethyl-2-oxocyclopentanecarboxylate, was identified as an optimal fragmental template capable of forming an imprinted polymer with molecular recognition properties towards aflatoxin B1 [57]. In this case, the fragmental template simulated only part of aflatoxin’s rings, D and E, with the loss of the planar conformation typical of coumarin templates, but its molecular structure makes it probable that the hydrogen bond interactions characteristic of coumarin templates are nevertheless fully preserved. 

**Figure 11:**
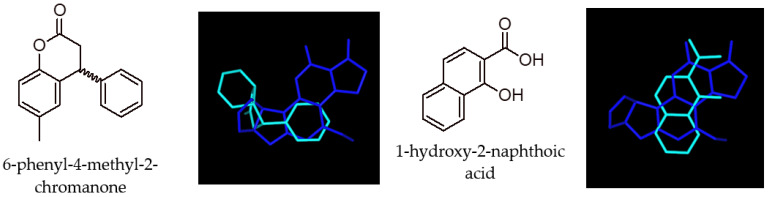
Non-conventional fragmental templates for aflatoxin imprinting. In the inserts: the superposition of AFB1 (blue) with (cyan) 6-phenyl-4-methyl-2-chromanone and 1-hydroxy-2-naphthoic acid.

In addition to the use of templates coinciding with structural fragments of the target molecule, in the case of aflatoxins, it must be noted that MIPs have also been successfully prepared using non-conventional templates that are structurally very different from the target. Song et al. reported the use of 6-phenyl-4-methyl-2-chromanone [58], where, although the chromanone nucleus actually corresponds to an aflatoxin fragment coinciding with the C-D rings, the presence of a phenyl group in position 6 constitutes a significant deviation from the very concept of a fragmental template. Similarly, Palmieri et al. used 1-hydroxy-2-naphthoic acid (HNA) as a fragmental template [59], taking the concept of the fragmental template itself to the limit, as HNA shares with the molecular structure of aflatoxins almost only the flatness of the central rings, as can be seen from Figure 11. 

**Figure 12:**
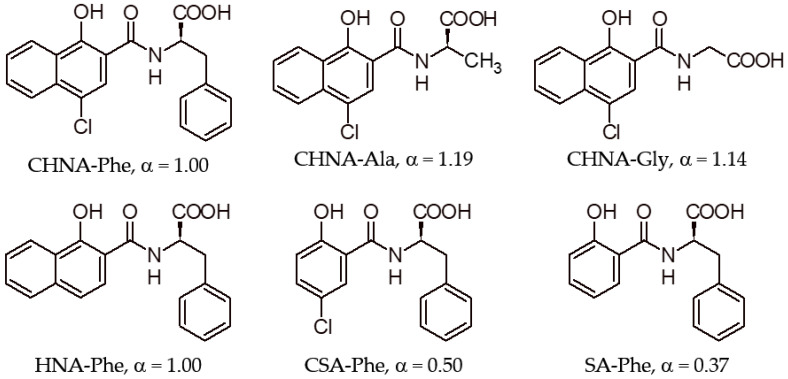
Molecular structures of fragmental mimics for ochratoxin A with measured α, relative binding affinity for OTA. CHNA-Phe: N-(4-chloro-1-hydroxy-2-naphthoylamido)-(L)-phenylalanine; CHNA-Ala: N-(4-chloro-1-hydroxy-2-naphthoylamido)-(L)-alanine; CHNA-Gly: N-(4-chloro-1-hydroxy-2-naphthoylamido)-glycine; HNA-Phe: N-(1-hydroxy-2-naphthoylamido)-(L)-phenylalanine; CSA-Phe: N-(4-chlorosalicylamido)-(L)-phenylalanine; and SA-Phe: N-salicylamido-(L)-phenylalanine.

With reference to the well-known CHNA-Phe mimic template, the fragmental approach was used to search for a template with the simplest possible molecular structure able to raise an imprinted polymer with binding properties towards ochratoxin A [60]. The experimental results were found to be compatible with in silico simulations of the complexation between the template molecules and the functional monomer methacrylic acid, showing that the simplification of the amino acidic sub-structure (L-alanine or glycine instead of L-phenylalanine) or elimination of the chlorine atom on the naphthalene ring system did not affect the molecular recognition of ochratoxin A, while the presence of the bulky naphthalene sub-structure in the template structure was necessary to preserve the molecular recognition effect (Figure 12).

### 2.3. The Solid-Phase Polymer Synthesis Approach

**Scheme 1:**
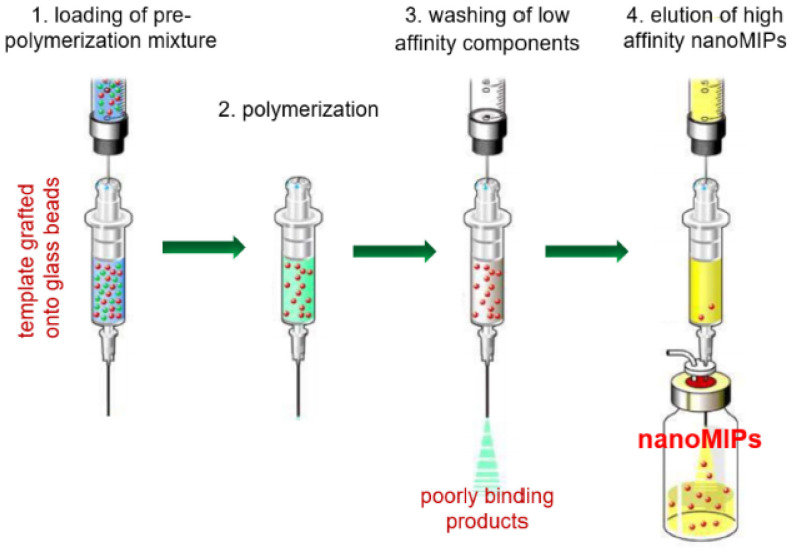
Representation of the solid-phase synthesis method. Different colours are related to different stage of the process [64].

The solid-phase polymer synthesis (SPPS) approach—illustrated in Scheme 1—is relatively more recent, as it was introduced in 2013 [61,62]. It uses solid-support glass beads covalently grafted with template molecules as a support for the polymerization process taking place in the interstitial space between the non-porous beads. The growth of cross-linked polymeric chains near the glass surface results in the imprinting of the nascent nanoparticles onto the grafted template molecules, producing high-affinity imprinted nanoparticles (nanoMIPs). Because of the strength of the non-covalent interactions between nanoparticles and template molecules, at the end of the polymerization process, any residual monomers, polymerization by-products, and low-affinity polymers can be easily removed by gentle washing, while high-affinity nanoMIPs can be recovered later by washing with a solution capable of breaking the non-covalent molecular interactions.

The SPPS approach has many advantages over traditional solution synthesis techniques, which make it potentially very useful in the preparation of MIPs against mycotoxins for analytical applications [63]. In fact, because the template molecules are covalently grafted onto the glass beads, no residual template molecules are present in nanoMIPs, avoiding the bleeding effect that affects other imprinted materials. Moreover, functionalized glass beads can be cleaned and reused many times, with an impact on the costs of synthesis, as this allows for the use of expensive molecules, while, in the case of mycotoxins, confinement on the glass surface eliminates any health risks from the residual template during the recovery step of the imprinted nanomaterial.

**Figure 13:**
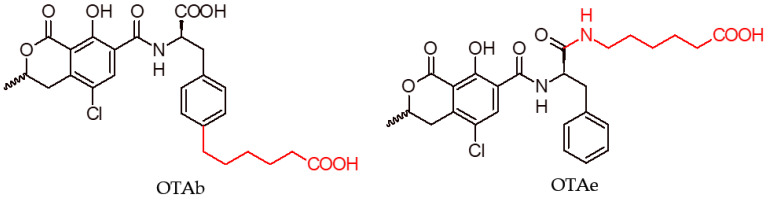
Molecular structures of modified ochratoxin A for grafting on aminated solid supports. In red, the added spacer arm.

It must be noted that grafting the templates onto a solid support necessarily requires the use of a molecule modified with a spacer arm as a template. In fact, structural modifications are not needed to differentiate it from the target compound or avoid unwanted molecular characteristics (i.e., toxicity, costs, and so on), but mainly to achieve stable grafting. This involves using the same strategies that are commonly used to prepare immunogens through the covalent conjugation of carrier proteins and target molecules, appropriately modified with a spacer arm. This approach has been reported for ochratoxin A, where it was possible to verify that, by positioning a spacer arm away from the functionalities upon which molecular recognition depends, the *p*-position on the phenylalanine ring (OTAa) and the carboxyl of the ochratoxin-α fragment (OTAe), respectively (Figure 13), the mimic template was able to induce the formation of nanoMIPs capable of effectively binding ochratoxin A [65]. A similar result has been reported more recently, where it was shown that even a mimic template structurally different from ochratoxin A (i.e., CHNA-Phe) is capable of producing nanoMIPs with good molecular recognition properties, without requiring the ad hoc addition of a spacer arm [64].

**Figure 14:**
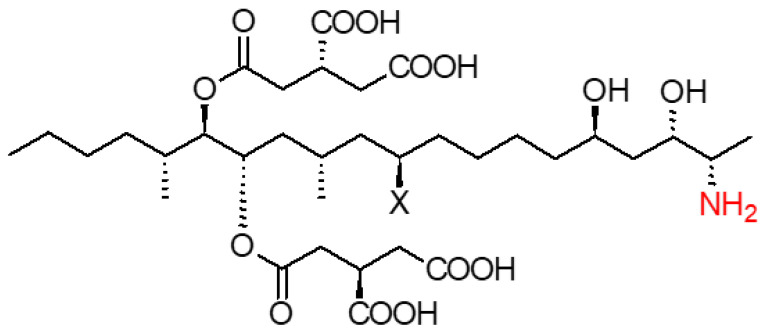
Molecular structure of fumonisins (B1: X = OH; B2: X = H). In red, the amino substituent used to synthetize covalent conjugates with glass beads.

The imprinting of fumonisins represents another interesting application of the SPPS approach to mycotoxins [66]. Because of their peculiar structure (Figure 14), no mimic/fragmental template has been reported in the literature to date; thus, fumonisin B1 and B2 were conjugated to a glutaraldeyhde-functionalized solid support by direct coupling via the amino function. The resulting nanoMIPs were used to develop MIP-based immunoassays, whose performance was found to be comparable with the corresponding antibody-based assays [67].

It is therefore possible to conclude that, although no extraction methods based on nanoMIPs have been published at the moment, the clear advantages of this approach make it a potential valid alternative to the use of mimic templates. 

## 3. Use of MIPs in Sample Preparation for Mycotoxin Detection

Depending on the analytical task, the advantages offered by MIPs can combine the selective separation and preconcentration of the target mycotoxin from the bulk matrix. They have been applied mainly in food matrices using different polymerization techniques, supporting substrates, selectivity, and analytical applicability. As described in detail in the following sections, MIPs have been used to develop several extraction methods, including off-line and on-line SPE (Solid-Phase Extraction), MSPE (Magnetic Solid-Phase Extraction), and SBSE (Stir Bar Sorptive Extraction). 

### 3.1. Solid-Phase Extraction with Commercial MIPs

At present, despite extensive matrix cleanup and target analyte preconcentration before analysis frequently being necessary, non-selective SPE sorbents (e.g., reverse-phase and ion-exchange, etc.) are still largely used in mycotoxin analysis because they are more easily accessible at lower prices [18]. In any case, commercial MIP cartridges for several mycotoxins are available on the market [68,69], and, in this context, examples of selective MISPE (molecularly imprinted solid-phase extraction) are reported in the literature for fumonisins B1-B3 in wheat and maize [70], ochratoxin A in ginger [71], grape juice, red wine and beer [72,73], roasted coffee [73], chili [73], and cocoa beans [74], patulin in apple juice, puree, and jam [75], and zearalenone in wheat and maize [76], vegetal oils [77] and beer [78] (Table S1). 

It must be considered that some key features of these commercial polymers, such as their composition, synthesis method, and the potential use of a mimic template, are information that is undisclosed by the manufacturers, but, as their physical appearance is that of a homogeneous powder, it is reasonable to assume that these polymers are produced through a scalable technique, probably emulsion or precipitation polymerization, and not through a bulk process. As for the nature of the template, obvious considerations for the cost of the production of the materials and the absence of any trace of target analytes in blank matrices suggest the use of mimic templates.

All reported cases show that HPLC methods preceded by a clean-up and pre-concentration step performed on MISPE cartridges present analytical performances comparable to, if not better than, those obtained with non-selective or immunoextraction supports. As significant examples, a study published in 2013 compared MISPE cartridges for ochratoxin A with other commercial solid-phase extraction media, such as immunoaffinity columns (IAC), Mycosep™ 229, Mycospin™, and Oasis^®^ HLB [73]. A total of 120 samples (30 wines, 30 beers, 30 roasted coffees, and 30 chili) were analyzed, and analytical recovery, reproducibility, limit of detection, and limit of quantification were evaluated. All the supports demonstrated suitability for OTA analysis, meeting the requirements specified in the EU regulations, with a moderate prevalence of IAC for wine and beer with an analytical recovery of >90%, as well as Mycosep™ for wine and chili, but MISPE was the most appropriate for coffee. In a more recent study [79], commercial MIPs for ochratoxin A and zearalenone mixed with a home-made citrinin-imprinted bulk material were used to prepare multi-target analyte MISPE cartridges. A comparison in terms of analytical recovery and cleanup efficiency with cartridges packed with several synthetic nano- and microfibers, graphene-doped composites, and restricted access materials showed that, in the extraction of mycotoxin from oat and rice milk MISPE, poly-caprolactone nanofibers and C_18_-restricted access cartridges performed better than the other solid phases in terms of cleanup efficiency, linearity, accuracy, and repeatability, with quantitative analytical recoveries for MISPE and poly-caprolactone nanofibers. 

### 3.2. Solid-Phase Extraction with Home-Made MIPs

Despite the progress made in the last twenty years in terms of synthetic approaches to MIPs with the aim of improving both their structural and morphological characteristics and bonding properties [80,81], the older technique of bulk onto polymerization still seems to prevail for home-made MIPs towards mycotoxins, with some relevant exceptions concerning miniemulsion polymerization for aflatoxin B1 [82], precipitation polymerization for citrinin [83], and surface grafting for aflatoxins [53,54,58,84,85], ochratoxin A [86], patulin [87], and zearalenone [88,89,90]. In almost all cases, polymers are prepared using mimic templates, with some notable exceptions in the cases of fumonisins [91] and T-2 toxin [92], for which, as previously underlined, no mimic templates are known in the literature.

The number of mycotoxins and the type of real matrices for which the development of extraction procedures based on home-made MIPs is reported in the literature is wider than that relating to commercially available MISPE cartridges, which demonstrates the continuing interest of the scientific community in this topic (Table S2). Aflatoxins B1, B2, G1, and G2 have been extracted from soy sauce and vinegar [53], wheat and corn [54,84,85], peanuts [54,58,82,85,93], rice [54,84,85], soybeans [54,84], medicinal herbs and spices [59], barley, beer and peanut oil [82], figs, hazelnuts, and red pepper [94]. Alternariol and alternariol 9-monomethylether have been extracted from tomato [36,37], tomato juice and sesame oil [37], maize, sunflower, and olive oils [95]. Citrinin has been extracted from rice [34,83], maize [35,83], and rye [83]. Fumonisins have been extracted from bell pepper, corn flakes, and rice [91]. Ochratoxin A has been extracted from soybeans [86] and red wine [95,96]. Patulin has been extracted mainly from apple juice [39,41,42,87,97], but also from apple, hawthorn, red wine, and tomato [95]. T-2 toxin has been extracted from barley, maize, and oat [92]. Zearalenone has been extracted from barley and rye [29], rice [29,89], wheat [29,88,89], corn [30,89,90], oats [88], maize, and sunflower and olive oils [95]. 

As in the case of MISPE developed on commercial cartridges, all those developed on home-made materials present analytical performances comparable to those obtained with non-selective or immunoextraction supports. For example, a bulk polymer imprinted with the T-2 mycotoxin was used to develop MISPE methods for maize, barley, and oat before HPLS-MS analysis [92]. A comparison with extraction protocols based on Oasis^®^ HLB or immunoaffinity chromatography (IAC) cartridges showed that, although the highest analytical recoveries were obtained with the Oasis^®^ HLB sorbent (74–104% vs. 60–73% for MISPE and 60–85% for IAC, respectively), MISPE and IAC were superior regarding selectivity, cross-reactivity, and the matrix effect. Limits of detection (LOD) and limits of quantification (LOQ) resulted in being lower for MISPE (LOD: 0.4–0.6 ng/kg and LOQ: 1.4–1.9 ng/g) in comparison with Oasis^®^ HLB (LOD: 0.9–3.5 ng/g and LOQ: 3.1–11.7 ng/g) and IAC (LOD: 0.3–2.3 ng/g and LOQ: 1.0–7.7 ng/g). Interestingly, no template bleeding was observed, making the use of a hypothetical mimic template unnecessary. 

### 3.3. On-Line Solid-Phase Extraction

**Scheme 2:**
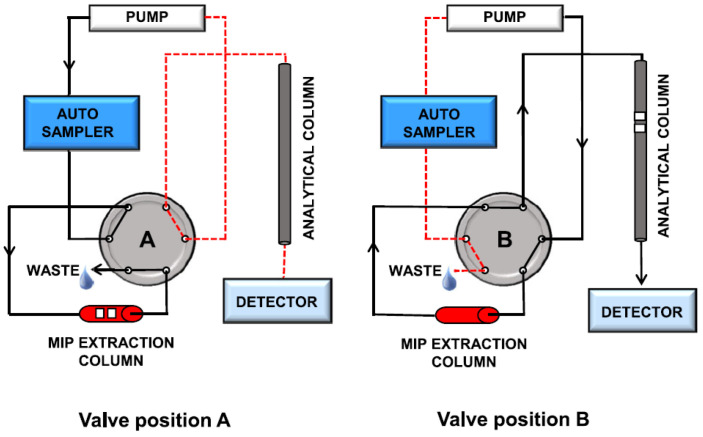
An on-line SPE system for mycotoxin extraction and determination. Valve in position A; mycotoxin clean-up and preconcentration. Valve in position B: mycotoxin separation on analytical HPLC column [99].

A drawback of off-line SPE is that it can be time-consuming, frequently requiring many steps before the HPLC analysis. On the contrary, on-line SPE (Scheme 2) offers several advantages, reducing the sample preparation time and thus increasing the sample throughput. Moreover, analytical precision and accuracy increase due to the absence of a sample evaporation step before injection in the analytical column. A higher sensitivity is also achieved in on-line SPE due to the analysis of the full extracted sample in contrast to off-line SPE, where only a fraction of the extract is injected into the analytical column [98]. It is therefore no coincidence that this approach has been successfully used for the extraction of several mycotoxins before HPLC analysis (Table S3).

Ochratoxin A is a target analyte towards which numerous examples of on-line SPE have been reported. In this case, the typology of the imprinted stationary phases appears to be quite varied and includes stainless steel frits grafted with a thin layer of electropolymerized polypyrrole [100,101,102] and an imprinted monolith prepared in a bare fused-silica capillary [103] or a HPLC microcolumn [104], but also traditional HPLC pre-columns packed with an MIP prepared via bulk polymerization [104,105]. In all these cases (except [105], where ochratoxin A is directly quantified without any further separative step), as the extraction step is followed by separation on an HPLC analytical column, the critical step for a successful method consists of the use of a desorption eluent compatible with the analytical eluent used in the HPLC column. This step therefore involves methanol/triethylamine as the desorption element and methanol/ammonium buffer as the analytical eluent [100,101,102], methanol/acetic acid as both the desorption and analytical eluent [103], methanol/acetic acid as the desorption element, and acetonitrile/water/acetic acid as the analytical eluent [104,105,106]. The on-line extraction of beer [103], wheat extracts [105,106], and red wine [100,101,102] resulted in being selective for the target analyte and allowed for the quantification of the contamination levels in these matrices being well below the legal limits established by the health authorities. Aside from ochratoxin A, on-line SPE in a packed microcolumn has been reported for citrinin in red yeast rice extracts [107] and patulin in apple juice [108,109]. It must be noted that, in the latter paper, the authors compared off-line and on-line SPE methods on the same imprinted polymer of commercial origin, concluding that, although on-line SPE leads to significant time savings, fewer human errors, and requires no handling of toxic solvents, it showed a worse LOD (15 versus 6 ng/mL), worse analytical recovery values (68.3–123.5 versus 81.2–109.9%), and a worse efficiency throughout the entire clean-up process in comparison with the off-line SPE. 

### 3.4. Dispersive Solid-Phase Microextraction

In dispersive solid-phase microextraction (DSPME), the solution containing the target analytes is not percolated through a cartridge containing the sorbent, as in the case for SPE, but it is the sorbent that is added into the sample solution and stirred for a specified time until the adsorption of the target analytes reaches its equilibrium. Then, the mixture is centrifuged or filtered to separate the sorbent, and the sample solution is discarded. A desorption solvent is finally added to the sorbent and a small volume of it is injected into the detection system for the determination of the target analytes [109]. In comparison with the similar Solid-Phase Microextraction (SPME), DSPME presents several advantages due to the absence of the fiber supporting the solid phase, including drawbacks due to the necessity of covalently grafting the sorbent onto the fibers, the fiber mechanical fragility, and the need to have the appropriate desorption apparatus connected to the analytical instrument [110].

Concerning mycotoxin analysis (Table S4), Fan et al. used bulk polymerization in the presence of 7-ethoxycoumarin to set-up the DSPME of aflatoxin B1 in peanut samples with detection via post-extraction Surface-Enhanced Raman Spectroscopy (SERS) with an LOD of 0.1 ng/mL [55]. Jayasinghe et al. used DSPME to extract aflatoxins B1, B2, G1, G2, and M1 from fish samples using a mimic-imprinted bulk material before an HPLC-MS/MS analysis [111]. Despite a strong matrix effect requiring the application of a standard addition method for aflatoxins G1 and M1, the extraction method was found to be simple, rapid, and highly selective, with analytical recoveries in the range of 80–100% and a good LOD (B1: 0.11 ± 0.03 ng/mL, B2: 0.20 ± 0.06 ng/mL, G1: 0.12 ± 0.03 ng/mL, G2: 0.20 ± 0.06 ng/mL, and M1: 0.10 ± 0.03 ng/mL, respectively) and LOQ (B1: 0.37 ± 0.11 ng/mL, B2: 0.67 ± 0.20 ng/mL, G1: 0.40 ± 0.12 ng/mL, G2: 0.68 ± 0.20 ng/mL, and M1: 0.32 ± 0.06 ng/mL, respectively). The same approach has been very recently reported by Thati et al. for the HPLC analysis of four aflatoxins (B1, B2, G1, and G2) in a large variety of food samples such as cereal grains, dry nuts, spices, oil seeds, vegetables, mushrooms, pulses, milk, and bread [112]. The extraction method proved to be compatible with a wide range of different matrices, highly selective, and reusable, with analytical recoveries in the range of 79.1–109.4% and a good LOD (B1: 0.193 ng/mL, B2: 0.087 ng/mL, G1: 0.208 ng/mL, and G2: 0.059 ng/mL, respectively) and LOQ (B1: 0.644 ng/mL, B2: 0.292 ng/mL, G1: 0.694 ng/mL, and G2: 0.197 ng/mL, respectively).

**Scheme 3:**
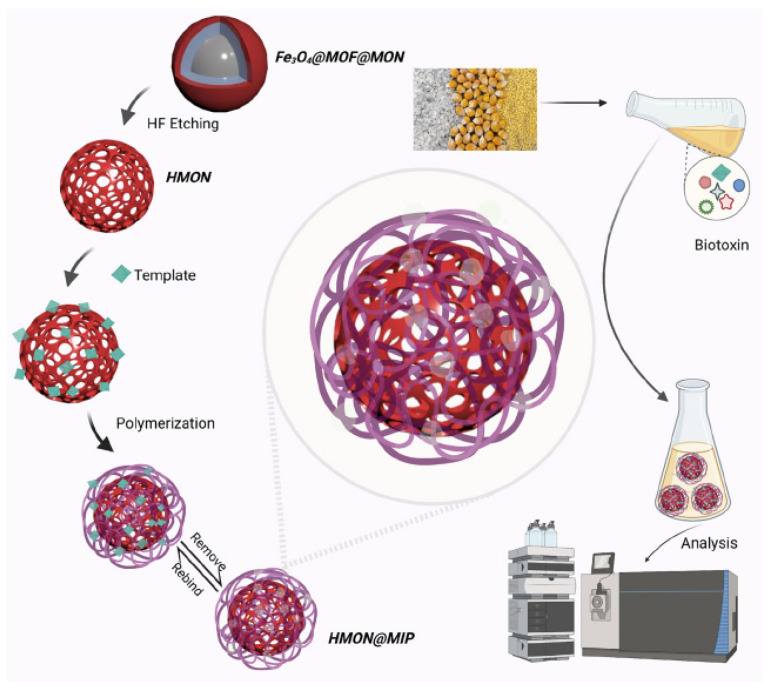
Schematic protocol of the integration of hollow-structured HMON@MIPs as adsorbents for DMSPE and highly sensitive determination of mycotoxins in food samples [113].

With the aim of increasing adsorption capacity, Yang et al. used a hollow-structured microporous organic network (HMON, Scheme 3) as a nanostructured scaffold to support and facilitate the imprinting of polymeric nanoparticles with mimic templates for aflatoxin B1 and sterigmatocystin [113]. The HMON@MIPs resulted in being characterized by a significantly increased imprinted binding site density and binding affinity for the target compounds. They were used for the extraction of mycotoxins from rice, maize, and soybean samples in DMSPE mode, resulting in being selective with analytical recoveries in the range of 81.2–95.1% and a very low LOD (4.4 and 6.7 pg/mL) and LOQ (14.6 and 23 pg/mL) for aflatoxin B1 and sterigmatocystin, respectively.

**Scheme 4:**
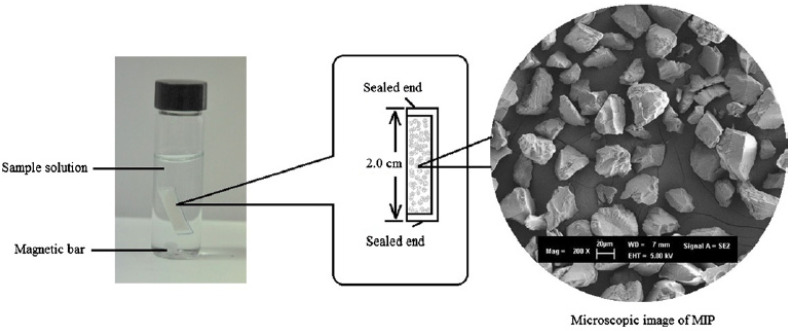
Schematic diagram and microscopic image of micro-solid phase extraction used by Lee et al. for ochratoxin A extraction and preconcentration [114].

Related to DSPME is the micro-solid-phase extraction method described by Lee et al. for the extraction of ochratoxin A from coffee and grape juice [114]. The authors packed an MIP prepared in bulk within a sealed polypropylene porous membrane, and the envelope was put into a vial containing the sample to be extracted (Scheme 4). After the extraction step, the ochratoxin was desorbed and analyzed via HPLC with analytical recoveries ranging from 90.6% to 101.5% and an LOD (0.06, 0.02, and 0.02 ng/mL) and LOQ (0.19, 0.06, and 0.08 ng/mL) comparable with those obtained via MISPE. The same approach was described by Chmangui et al. for the extraction of aflatoxins B1 and B2 from seed-derived beverages (soya, rice, almond, coconut, oat, and tigernut) [52]. Despite a marked matrix effect, aflatoxins were determined via HPLC-MS/MS with analytical recoveries in the range of 91–104% and limits of detection within the 0.085–0.207 ng/mL range. 

### 3.5. Magnetic Solid-Phase Extraction and Stir Bar Sorptive Extraction

MSPE is based on the dispersion of paramagnetic nano- or microparticles coated with a sorbent layer in a sample solution. Once the retention process of the target analytes is concluded, the paramagnetic particles are separated from the solution by the application of an external magnet and the supernatant is then discarded. Subsequently, the elution process is carried out through the addition of an appropriate solvent to ensure the analytes’ desorption [115,116]. In addition to its operational simplicity, MSPE is efficient, economical, and does not need additional steps, such as centrifugation, precipitation, or filtration, thus avoiding the loss of analytes. Moreover, the ability of limited amounts of particles—typically tens of mg—to extract target analytes from large-volume samples allows for easily obtaining high preconcentration factors, greatly amplifying the analytical sensitivity of the methods.

In regard to the analysis of mycotoxins (Table S5), if compared to off- and on-line SPE, the examples of MSPE reported in the literature are relatively more recent, no older than about ten years ago and concerning a smaller number of target analytes and real matrices. Aflatoxins B1, B2, and G2 have been extracted jointly from corn [56], B1 and B2 from corn and peanut oil [117], and B1 alone from barley and beer [118] and bovine liver [119]. Using deoxynivalenol as a template, fusarotoxins (fusarenon-X, deoxynivalenol, 3-acetyldeoxynivalenol, 15-acetyldeoxynivalenol, T-2 toxin, and HT-2 toxin) have been extracted from rice [120]. Ochratoxins A, B, and C have been extracted from rice and wine [121]. Patulin has been extracted from several fruit juices [44,122]. Sterigmatocystin has been extracted from wheat [123]. To conclude, zearalenone has been extracted from a wider variety of matrices: buckwheat [32], corn [124,125,126], corn oil [127], flour [124], maize [32,128], millet [126], rice [32,123,124], and wheat [32,125,129]. In all these cases, MSPE methods present analytical performances in terms of limits of detection and quantification, analytical recovery, and matrix clean-up comparable to, if not better than, those obtained with cartridge-based MISPE methods. As a relevant example, the MSPE for fusarotoxins reported by Pan et al. [120] shows, for T2-toxin, a limit of detection of 5 pg/g and a limit of quantification of 20 pg/g with HPLC-MS/MS detection, while the off-line MISPE for the T2-toxin reported by De Smet et al. [92] shows limits of detection and quantification higher than two orders of magnitude, 0.4–0.6 ng/g and 1.4–1.9 ng/g, respectively. 

**Scheme 5:**
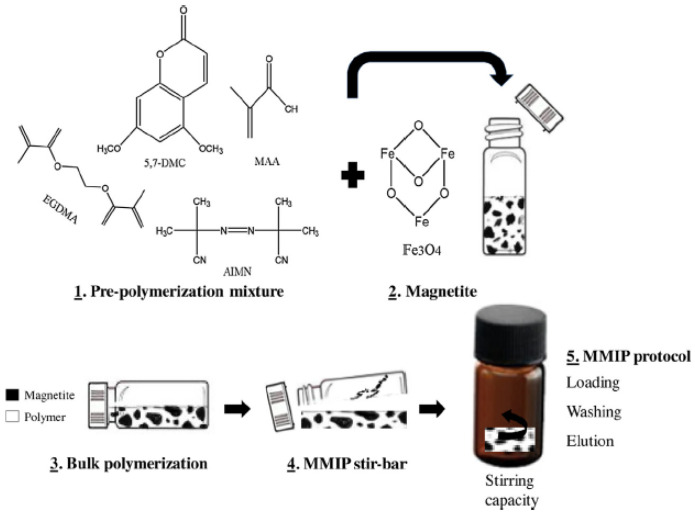
Preparation and use of magnetic molecularly imprinted stir-bars [50].

A variant of the MSPE is the SBSE, where, instead of paramagnetic nano- or microparticles, a solid magnetic stir bar grafted with a layer of sorbent material is used to extract the target analytes [130]. In regard to mycotoxin analysis, an interesting example of SBSE concerns the development of magnetic stir bars composed of a monolithic MIP embedding paramagnetic Fe_3_O_4_ microparticles through a bulk polymerization process (Scheme 5). When imprinted with a mimic for aflatoxins [50], the stir bar was able to extract aflatoxin M1 from milk powder for baby food with an analytical recovery of 60% (LOD: 1.0 ng/kg and LOQ: 0.3 ng/kg) and aflatoxins B1, B2, G1, and G2 in cereal-based baby foods with analytical recoveries of 43, 40, 44, and 39%, respectively (LOD: 0.9, 0.7, 1.0, and 1.7 ng/kg and LOQ: 3.0, 2.3, 3.5, and 5.8 ng/kg). Concerning patulin [43], it was extracted from apple with recoveries of 60–70% (LOD: 10 ng/g and LOQ: 50 ng/g). 

## 4. Conclusions

As shown in this review, in the last fifteen years, the use of molecular imprinting technology to prepare efficient and selective sorbents for the clean-up and preconcentration of mycotoxins in complex samples has expanded significantly, both in terms of target analytes and extraction methods. One of the main issues that hindered the development of MIP-based sorbents, i.e., the difficulty of directly using target mycotoxins as templates, is currently largely resolved—except perhaps for fusarotoxins—thanks to the extensive use of easy to achieve, safer, and less expensive mimic templates. The appearance on the market of commercial products has demonstrated that molecular imprinting technology is mature, and that the MIP-based sorbents can compete effectively with traditional solid-phase extraction materials in terms of selectivity and with immunoaffinity extraction in terms of stability and the low cost of their preparation.

Nevertheless, focusing the attention on its main competitor technique, i.e., immunoaffinity extraction, there are far less papers concerning the MISPE of mycotoxins. There are some reasons for this apparent lack of interest. First of all, immunoaffinity extraction is an extremely valid competitor: it has the ability to clean-up heavily contaminated samples and it efficiently removes interfering substances because of the innate high binding selectivity of natural antibodies. As a consequence of this, over many years, immunoaffinity extraction has become a robust technique in mycotoxin analysis. Thus, even if imprinted materials are potentially competitive with immunoaffinity-based materials, the acceptance of this technology remains low. Generally speaking, the acceptance of new technology meets resistance, if not distrust, from users of older, consolidated methods (if this approach works well, why change?), and this has immediate consequences on the diffusion of MISPE methods.

However, it is our opinion that the continuous evolution of molecular imprinting technology, which is currently moving towards nanostructured materials with properties becoming increasingly close to those of natural antibodies, makes it likely that, in the coming years, extraction techniques based on MIPs will be able to evolve further, both in terms of improving techniques (increased selectivity, multiple targets, and a reduction in matrix effects) and in terms of analytical targets not yet taken into consideration (emerging and masked mycotoxins). 

## Data Availability

Not applicable.

## Supplementary Materials

The following supporting information can be downloaded at: https://www.mdpi.com/article/10.3390/toxins16010047/s1, Table S1: SPE of mycotoxins on commercial MIP cartridges; Table S2: SPE of mycotoxins on home-made MIP cartridges; Table S3: on-line SPE of mycotoxins; Table S4: DSPME of mycotoxins; Table S5: MSPE/SBSE of mycotoxins; and Figure S1: molecular structures of all the micotoxins considered in this paper.
