# Supplementary material for: Mycotoxins-Imprinted Polymers: A State-of-the-Art Review
Source: Toxins (Basel). 2024 Jan 15;16(1):47. doi: 10.3390/toxins16010047 (PMC10818578; doi:10.3390/toxins16010047)
Supplement: Supplementary file 1 [file toxins-16-00047-s001.zip › toxins-2766304-supplementary.pdf]

# Supplementary Materials: Mycotoxins-Imprinted Polymers: A State-of-the-Art Review

**Table S1.** SPE of mycotoxins on commercial MIP cartridges.

| Mycotoxin | Sample | Analytical method [1] | LOD [2] | LOQ [2] | % Recovery | Ref. |
|---|---|---|---|---|---|---|
| Fumonisins | wheat, maize | HPLC-MS | - | 25 | 95 - 100 | [70] |
| Ochratoxin A | ginger | HPLC-MS | 0.09 | 0.30 | 87.6 - 94.5 | [71] |
| | beer, grape, red wine | HPLC-FL | 0.025 | 0.08 | 91.6 - 101.7 | [72] |
| | wine | HPLC-MS | 0.14 | 0.48 | 70.3 - 80.4 | [73] |
| | beer | | 0.29 | 0.97 | 63 - 76 | |
| | coffee | | 0.08 | 0.29 | 89.2 - 84.8 | |
| | chili | | 1.77 | 5.93 | 81.0 - 83.8 | |
| | cocoa | HPLC-FL | 0.62 | 1.25 | 86.5 | [74] |
| Patulin | apple juice, puree, jam | HPLC-UV | 0.6 | - | 77 - 96 | [75] |
| Zearalenone | wheat, maize | HPLC-FL | - | - | 82 - 90 | [76] |
| | vegetable oils | HPLC-FL | 1.2 | 4 | > 72 | [75] |
| | beer | HPLC-FL | 1.5 | - | 99 - 100.1 | [76] |

[1] HPLC-FL: fluorescence detection; HPLC-MS: mass spectrometric detection; HPLC-UV: ultraviolet detection. [2] ng/g or ng/mL

**Table S2.** SPE of mycotoxins on home-made MIP cartridges.

| Mycotoxin | Template | Polymer | Sample | Analyte | Analytical method [1] | LOD [2] | LOQ [2] | % Recovery | Ref. |
|---|---|---|---|---|---|---|---|---|---|
| Aflatoxins | 5,7-dimethoxycoumarin | surface imprinting on inverse photonic crystals | soy sauce, vinegar | B1 | HPLC-FL | 0.4 | - | 73 -92 | [53] |
| | 7-acetoxy-3-methylcoumarin | surface imprinting on FDU-12 silica | wheat, rice, corn, peanut, soybean | B1, B2, G1, G2 | HPLC-FL | - | 0.15 - 0.2 | 82.6 - 116.7 | [54] |
| | 6-phenyl-4-methyl-2-chromanone | surface imprinting on SBA-15 silica | peanuts | B1 | HPLC-FL | 0.118 | 0.393 | 79.5 - 91.2 | [58] |
| | hydroxy-2-naphthoic acid | bulk | medicinal herbs, spices | B1, B2, G1, G2 | HPLC-MS | 0.005 - 0.027 | 0.02 - 0.09 | 60 - 90 | [59] |
| | B1 | miniemulsion | barley, beer, peanuts, peanut oil | B1, M1 | HPLC-FL | - | 0.05 - 0.16 | 83 - 96 | [82] |
| | quercetin | surface imprinting on MOF UiO-66 | corn, rice, soybeans, wheat | B1, B2, G1, G2 | HPLC-FL | 0.09 - 0.13 | - | 74.3 - 98.6 | [84] |
| | 5,7-dimethoxycoumarin | surface imprinting on quantum dots | corn, peanuts, rice, wheat | B1, B2, G1, G2 | HPLC-FL | 0.03 - 0.05 | 0.1 - 0.2 | 98.9 - 119.7 | [85] |
| | B1, B2, G1, G2 (mixture) | bulk in cryogel | figs, hazelnuts, peanuts, red pepper | B1, B2, G1, G2 | HPLC-FL | - | 0.025 | - | [93] |
| Alternariol | THDP | bulk | tomato | ALT | HPLC-FL | - | - | 81 - 103 | [36] |
| | THDP | bulk | tomato juice, sesame oil | ALT, AME | HPLC-MS | - | 1.1 - 2.8 | 92.5 - 106.2 | [37] |
| | THDP (mixed with CHDB) | bulk in sacrificial silica beads | maize, sunflower and olive oils | ALT | HPLC-FL | - | 2 | 92 - 113 | [94] |
| Citrinin | 2-DHNA | bulk | rice | CIT | HPLC-UV | 0.5 | 1.5 | 85.9 - 98.8 | [34] |
| | 2-DHNA | bulk | maize | CIT | HPLC-FL | 10 | 30 | 82.3 - 91.5 | [35] |
| | CIT | precipitation | rye | CIT | HPLC-UV | 0.35 | - | 98 - 100.0 | [83] |

| | | | | | | | | | |
|---|---|---|---|---|---|---|---|---|---|
| Fumonisins | FB1 | bulk | bell pepper, corn flakes, rice | FB1, FB2, FB3 | HPLC-MS | 4.5 - 22 | 9 - 44 | 62 - 83 | [91] |
| Ochratoxin A | CHNA-Phe | surface imprinting on MOF MIL-53 | soybeans | OTA | HPLC-FL | 0.04 | 0.12 | 79 - 106 | [86] |
| | CHNA-Phe | bulk | red wine | OTA | HPLC-FL | 0.01 | 0.033 | > 90 | [95] |
| | CHNA-Phe | bulk | red wine | OTA | HPLC-FL | 0.075 | 0.225 | 88 -102 | [96] |
| Patulin | 2-hydroxynicotinic acid | bulk | apple juice | PAT | HPLC-UV | 10 | 33.3 | 60 - 66 | [39] |
| | oxyindole + 2-hydroxynicotinic acid | bulk | apple juice, apple, hawtorn, red wine, tomato | PAT | HPLC-MS | 0.05 - 0.2 | 0.2 - 0.5 | 81.3 - 106.3 | [41] |
| | 2oxindole | bulk | apple juice | PAT | HPLC-UV | 5 | 16 | 84.3 - 88.9 | [42] |
| | oxindole | surface imprinting on silica beads | apple juice | PAT | HPLC-UV | 10 | 40 | 90.1 - 96.5 | [87] |
| | PAT | ormosil | apple juice | PAT | HPLC-UV | 8.6 | 28.6 | 82 - 98 | [97] |
| T2-toxin | T-2 | bulk | maize, barley, oat | T-2 | HPLC-MS | 0.4 - 0.6 | 1.4 - 1.9 | 60 - 73 | [92] |
| Zearalenone | CHDB | bulk | barley, corn, rice, rye, wheat | ZAN, α-ZOL | HPLC-FL | 1.7 - 2.4<br>0.7 - 1.3 | - | 85 - 97 | [29] |
| | CHDB | surface imprinting in halloysite nanotubes | oats, wheat | ZEN | HPLC-FL | 0.5 - 1.67 | - | 77.1 - 102.4 | [88] |
| | CHDB | surface imprinting on MOF MIL-101 | corn, rice, wheat | ZEN | HPLC-FL | 2.09 - 4.16 | 6.25 - 12.50 | 81.7 - 90.1 | [89] |
| | CHDB | surface imprinting on hydroxyapatite | corn | ZEN | HPLC-FL | 1.32 | 4.38 | 70.1 - 101.9 | [90] |
| | CHDB (mixed with THDP) | bulk in sacrificial silica beads | maize, sunflower and olive oils | ZEN | HPLC-FL | - | 2 | 92 - 113 | [94] |

[1] HPLC-FL: fluorescence detection; HPLC-MS: mass spectrometric detection; HPLC-UV: ultraviolet detection. [2] ng/g or ng/mL

**Table S3.** on-line SPE of mycotoxins.

| Mycotoxin | Template | Polymer | Sample | Analyte | Analytical method [1] | LOD [2] | LOQ [2] | % Recovery | Ref. |
|---|---|---|---|---|---|---|---|---|---|
| Citrinin | 2-DHNA | bulk, packed in microcolumn | red yeast rice extract | CIT | HPLC-FL | 7.5 | 25 | 76 - 91 | [107] |
| Ochratoxin A | OTA | polypyrrole, grafted on stainless steel | red wine | OTA, OTB | HPLC-FL | 0.05 | - | 34 - 45 | [100] |
| | OTA | polypyrrole, grafted on stainless steel | red wine | OTA | HPLC-FL | 0.012 | 0.041 | - | [101] |
| | OTA | polypyrrole, grafted on stainless steel | red wine | OTA | HPLC-FL | 0.08 | - | - | [102] |
| | OTA | monolith, in silica capillary | beer | OTA | HPLC-FL | 0.07 | 0.2 | 82.8 | [103] |
| | OTA | monolith, in microcolumn | beer | OTA | HPLC-FL | 0.05 | - | 85.9 | [104] |
| | OTA | bulk, packed in microcolumn | wheat extract | OTA | fluorescence | 1.2 | - | 93.9 | [105] |
| | CHNA-Phe | bulk packed in microcolumn | wheat extract | OTA | HPLC-FL | 0.04 | 0.12 | 79.0 - 106 | [106] |
| Patulin | not disclosed | commercial polymer, in silica capillary | apple juice, cider | PAT | CZE-MS | 0.3 | 1 | 94 - 98 | [108] |
| | not disclosed | commercial polymer, in microcolumn | apple juice | PAT | HPLC-UV | 15 | 50 | 81.2 - 109.9 | [99] |

[1] HPLC-FL: fluorescence detection; HPLC-MS: mass spectrometric detection; HPLC-UV: ultraviolet detection; CZE-MS: capillary zonal electrophoresis – mass spectrometry. [2] ng/g or ng/mL

**Table S4.** DSPME of mycotoxins.

| Mycotoxin | Template | Polymer | Sample | Analyte | Analytical method [1] | LOD [2] | LOQ [2] | % Recovery | Ref. |
|---|---|---|---|---|---|---|---|---|---|
| Aflatoxins | 5,7-dimethoxycoumarin | bulk | seed-derived beverages | B1, B2 | HPLC-MS | 0.085 - 0.207 | - | 91 - 104 | [52] |
| | 7-ethoxycoumarin | bulk | peanuts | B1 | SERS | 0.1 | - | 93 - 102 | [55] |
| | 5,7-dimethoxycoumarin | bulk | fish | B1,B2, G1,G2, M1 | HPLC-MS | B1: 0.11, B2: 0.20, G1: 0.12, G2: 0.20, M1: 0.10 | B1: 0.37, B2: 0.67, G1: 0.40, G2: 0.68, M1: 0.32 | 80 - 100 | [111] |
| | 7-methoxycoumarin | precipitation | cereal grains, dry nuts, spices, oil seeds, vegetables, mushrooms, pulses, milk and bread | B1, B2, G1, G2, | HPLC-FL | B1: 0.193, B2: 0.087 G1: 0.208, G2: 0.059 | B1: 0.644, B2: 0.292, G1: 0.694, G2: 0.197 | 79.1 - 109.4 | [112] |
| | 5,7-dimethoxycoumarin | bulk in nanoscaffold | rice, maize, soybean | B1 | HPLC-FL | 4.4 | 14.6 | 81.2 - 95.1 | [113] |
| Ochratoxin A | not disclosed | bulk (commercial) | coffee, grape juice | OTA | HPLC-FL | 0.06, 0.02 | 0.19, 0.06 | 90.6 - 101.5 | [114] |
| Sterigmatocystin | crysazyn | bulk in nanoscaffold | rice, maize, soybean | STE | HPLC-FL | 6.7 | 23 | 81.2 - 95.1 | [113] |

[1] HPLC-FL: fluorescence detection; HPLC-MS: mass spectrometric detection; HPLC-UV: ultraviolet detection; CZE-MS: capillary zonal electrophoresis – mass spectrometry. [2] ng/g or ng/mL

**Table S5.** MSPE / SBSE of mycotoxins.

| Mycotoxin | Template | Polymer | Sample | Analyte | Analytical method [1] | LOD [2] | LOQ [2] | % Recovery | Ref. |
|---|---|---|---|---|---|---|---|---|---|
| Aflatoxins | 5,7-dimethoxycoumarin | stir bar composite | cereals, milk powder (M1) | B1, B2, G1, G2, M1 | HPLC-MS | 0.9, 0.7, 1.0, 1.7, 0.3 | 3.0, 2.3, 3.5, 5.8, 1.0 | 43, 40, 44, 39, 60 | [50] |
| | ethylcoumarin-3-carboxyate | grafting on $Co_3O_4$ | corn | B1 B2 G2 | HPLC-MS | 0.07 - 0.05 | 0.15 - 0.22 | 75.1 - 99.4 | [56] |
| | 5,7-dimethoxycoumarin | polydopamine on $Fe_3O_4$ | corn, peanut oil | B1, B2 | HPLC-FL | 0.0004 - 0.024 | - | 89.0 - 105.0 | [117] |
| | B1 | grafting on $Fe_3O_4$@Au | barley, beer | B1 | UV-Vis | 6.12 | 18.6 | 94.5 - 97.3 | [118] |
| | B1 | grafting on $Fe_3O_4$ | liver | B1 | HPLC-FL | 0.05 | - | 78 - 83 | [119] |
| Fusarotoxins | DON | grafting on $Fe_3O_4$ | rice | DON, 3-ADON, 15-ADON, FUS-X, T-2, HT-2 | HPLC-MS | 0.005 - 0.001 | 0.02 - 0.03 | 89.2 - 103.1 | [120] |
| Ochratoxin A | OTA | grafting on $Fe_3O_4$ | rice, wine | OTA, OTB, OTC | HPLC-FL | 0.0018, 0.018, 0.0032 | - | 71.0 - 88.5 | [121] |
| Patulin | oxindole | stir bar composite | apple | PAT | HPLC-MS | 10 | 50 | 60 - 70 | [43] |
| | oxindole | ormosil on $Fe_3O_4$ | apple, grape, orange and pear juices | PAT | HPLC-MS | 3 | 10 | 86.4 - 95.5 | [44] |
| | oxyindole + 2-hydroxynicotinic acid | polydopamine on $Fe_3O_4$ | apple, grape, and orange juices | PAT | HPLC-MS | 0.1 | - | 79.4 - 97.9 | [122] |
| Sterigmatocystin | crysazyn | grafting on $Fe_3O_4$ | wheat | STE | HPLC-UV | 0.63 | 1.4 - 1.9 | 87.6 - 96.9 | [123] |

| | | | | | | | | | |
|---|---|---|---|---|---|---|---|---|---|
| Zearalenone | quercetin | grafting on $Fe_3O_4$ | buckwheat, maize, rice, wheat | ZEN | HPLC-MS | 0.044 | 0.14 | 81 - 98 | [32] |
| | warfarin | ormosil on $Fe_3O_4$ | corn, flour, rice, | ZEN | HPLC-FL | 0.4 | 0.9 | 90.6 - 100 | [124] |
| | warfarin | ormosil on $Fe_3O_4$ | corn, rice, wheat | ZEN | HPLC-FL | 0.1 | 0.3 | 96.3 - 98.8 | [125] |
| | naringenin | grafting on hydroxyapatite - $Fe_3O_4$ | *coix lachryma*, corn, millet, | ZEN | HPLC-FL | 2 | 6.65 | 62.0 - 95.2 | [126] |
| | quercetin | polydopamine on $Fe_3O_4$ | corn oil | ZEN | HPLC-FL | 0.68 | - | 93.8 - 101.0 | [127] |
| | warfarin | grafting on halloysite - $Fe_3O_4$ | maize | ZEN | HPLC-FL | 2.5 | 8 | 74.9 - 88.4 | [128] |
| | quercetin | grafting on $Fe_3O_4$ | wheat | ZEN | HPLC-FL | 0.55 | - | 92.1 - 96.0 | [129] |

[1] HPLC-FL: fluorescence detection; HPLC-MS: mass spectrometric detection; HPLC-UV: ultraviolet detection. [2] ng/g or ng/mL

**Figure S1**: molecular structures of all the micotoxins considered in this paper

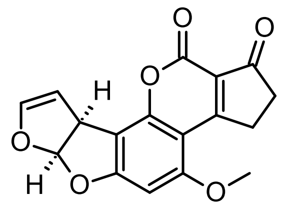

Aflatoxin B1, AFB1

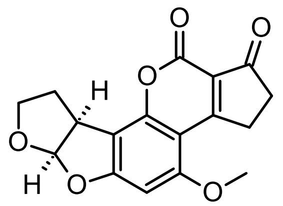

Aflatoxin B2, AFB2

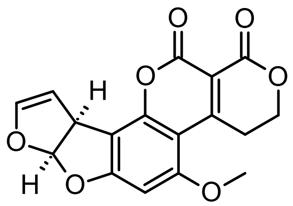

Aflatoxin G1, AFG1

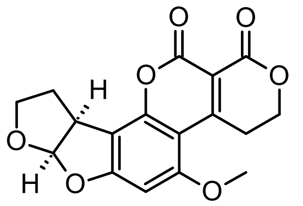

Aflatoxin G2, AFG2

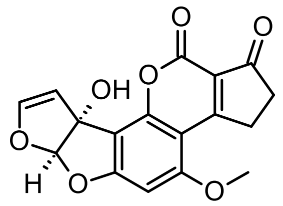

Aflatoxin M1, AFM1

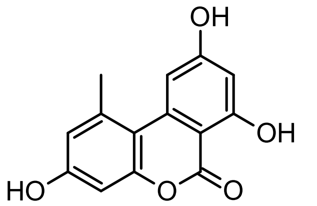

Alternariol, ALT

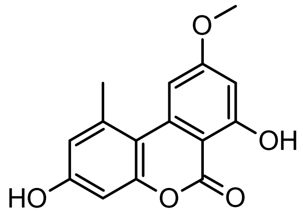

Alternariol 9-methylether, AME

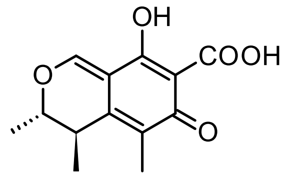

Citrinin, CIT

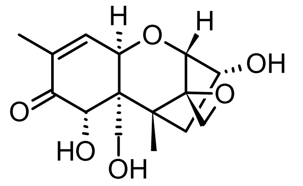

Deoxynivalenol, DON

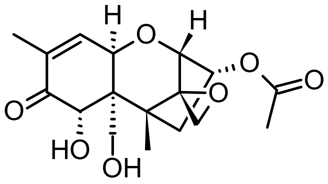

3-Acetyl-deoxynivalenol, 3-ADON

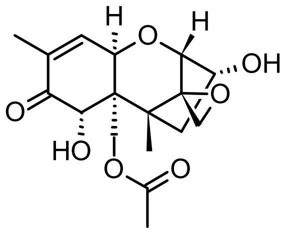

15-Acetyl-deoxynivalenol, 15-ADON

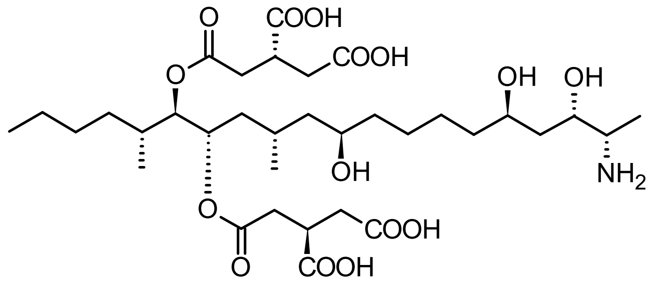

Fumonisin B1, FB1

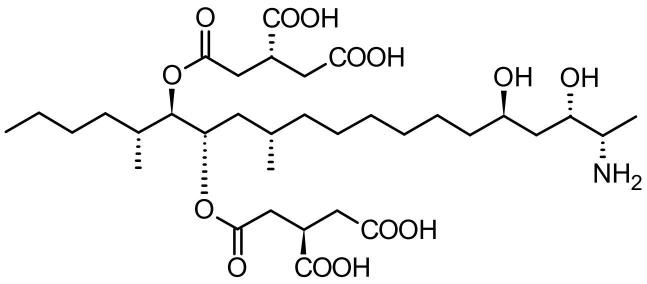
Fumonisin B2, FB2

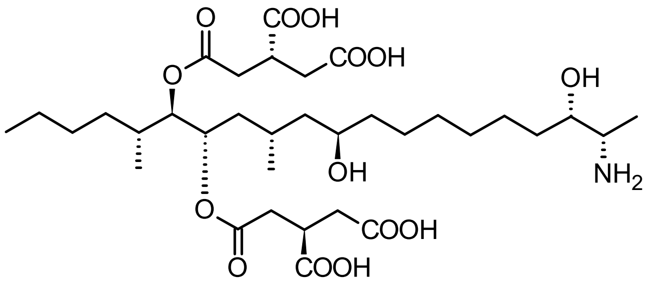
Fumonisin B3, FB3

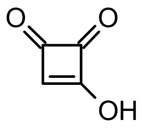
Moniliformin, MON

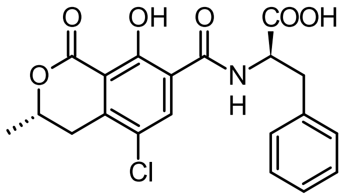
Ochratoxin A

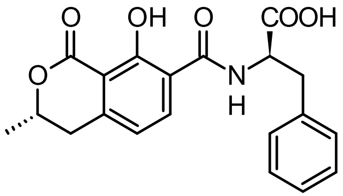
Ochratoxin B

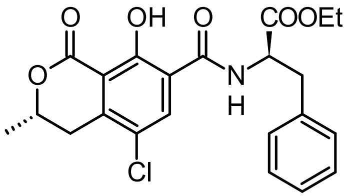
Ochratoxin C

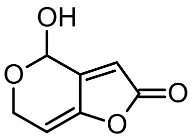
Patulin, PAT

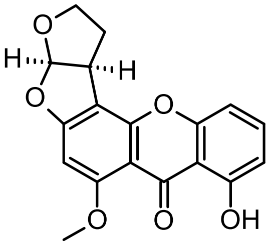
Sterigmatocystin, STE

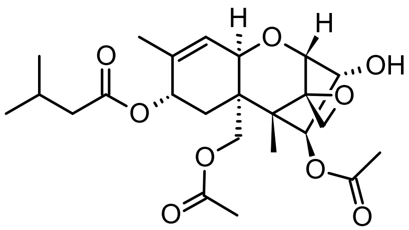
T-2 toxin, T2

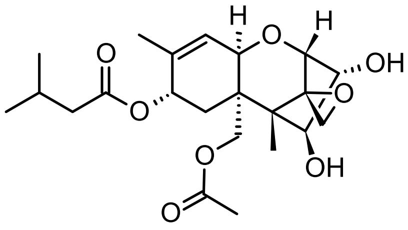
HT-2 toxin, HT2

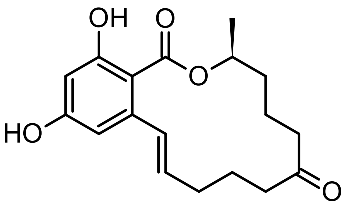
Zearalenone, ZON

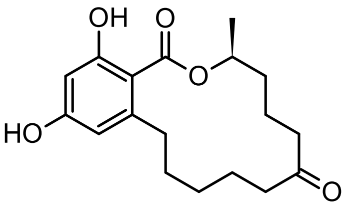
Zearalanone, ZAN
